# Shame norms, achievement pressure, and controlled self-regulation: cultural pathways to psychological distress and performance outcomes in endurance athletes

**DOI:** 10.3389/fpsyg.2026.1856560

**Published:** 2026-08-19

**Authors:** Benshu Zhu, Dingwu Liu, Xiaohan Chen

**Affiliations:** 1 School of Physical Education and Sport Science, QuFu Normal University, Jining, Shandong, China; 2 School of WuShu, HeNan University, Kaifeng, HeNan, China

**Keywords:** achievement pressure, ACT-informed psychological skills training, controlled self-regulation, endurance performance, psychological distress, self-determination theory, shame norms

## Abstract

Shame norms, defined as perceived group-level standards linking athletic failure to collective disgrace, represent a culturally specific but understudied antecedent of motivational regulation in Chinese competitive sport. The present study tested a dual-pathway model in which shame norms are associated with achievement pressure, which selectively relates to controlled self-regulation as a performance-related pathway while simultaneously contributing to psychological distress as a health-cost pathway. A total of 388 competitive long-distance runners were initially recruited from five universities and four running clubs across three Chinese cities, and 345 athletes who completed all five measurement waves constituted the final analytic sample. Participants completed measures of shame norms, achievement pressure, behavioural regulation, psychological distress, burnout, and social support at each wave, alongside a standardised 5,000 m time trial. An ACT-informed multimodal psychological skills training programme was delivered across Weeks 1 to 8. Baseline latent structural equation models supported the hypothesised associations: shame norms predicted achievement pressure, achievement pressure predicted controlled but not autonomous self-regulation, and shame norms and achievement pressure were associated with psychological distress; observed-score OLS regressions on composite scores showed a consistent pattern. Post-intervention path coefficients were attenuated, consistent with statistical range restriction and possible motivational decoupling, but the single-arm design prevents causal attribution to the programme. Overall, the findings identify shame norms as a distinct upstream antecedent in SDT-based sport psychology models and highlight the psychological costs of culturally driven controlled motivation in endurance athletes.

## 1 Introduction

Athletic performance at the elite level is shaped not only by physiological capacity and technical skill but also by the psychosocial environments in which athletes train and compete (Ntoumanis and Moller, 2025; Woolf et al., 2024). In East Asian sport cultures, Confucian values of collective harmony, hierarchical loyalty, and the preservation of face (mianzi) create a distinctive motivational context that differs substantially from the individualistic frameworks assumed by most Western sport psychology theories (Ho, 1976; Tsang, 2025). Within Chinese competitive sport in particular, failure is not merely a personal setback. It also carries implications for the social standing of one’s family, training group, and institution, embedding athletic participation in a web of normative shame expectations that extends well beyond the individual competitor (Ho, 1976; Woolf et al., 2024).

Research on shame in achievement contexts has predominantly focused on trait shame-proneness, defined as an individual’s chronic tendency to evaluate the global self negatively in response to failure (Tangney et al., 2007). However, trait-level shame is conceptually and empirically distinct from shame norms, which refer to the perceived social standards within a training group that link athletic failure to collective disgrace. This distinction matters because normative shame pressure operates at the level of the social environment rather than the individual disposition. Two athletes with identical shame-proneness profiles will experience different motivational and affective consequences if one trains in a group that trivialises failure and the other in a group in which failure is publicly shameful. Despite its theoretical importance, shame norms as a group-level contextual variable have received scant empirical attention in competitive sport, and, to the authors’ knowledge, no validated instrument has previously operationalised this construct independently of individual trait affect.

Self-determination theory (SDT; Ryan and Deci, 2000) offers a comprehensive framework for linking social-contextual pressures to motivational regulation and psychological outcomes in sport. SDT proposes that behaviour can be regulated along a continuum from controlled regulation, driven by external contingencies and introjected pressure, to autonomous regulation, characterised by identified and intrinsic motivation (Deci and Ryan, 2000). Controlled self-regulation (CSR), comprising external and introjected regulation, has been consistently associated with burnout, dropout, and compromised well-being (Madigan et al., 2022; Shannon et al., 2023), whereas autonomous self-regulation (ASR) is linked to sustained engagement, subjective vitality, and higher performance (Ntoumanis et al., 2021; Ntoumanis and Moller, 2025). A growing body of SDT-informed intervention research has confirmed these differential outcome profiles across diverse sporting populations (Reinebo et al., 2024; Shannon et al., 2023).

The present study integrates these theoretical traditions to propose a dual-pathway model of shame norms, motivational regulation, and athletic outcomes in Chinese competitive long-distance running. The performance pathway posits that shame norms elevate perceived achievement pressure (H1). In turn, achievement pressure selectively activates controlled rather than autonomous self-regulation (H2a: AP → CSR; H2b: AP → ASR expected null), which then predicts endurance performance (H3: SR → EP). The distress pathway posits that achievement pressure and shame norms together directly generate psychological distress and burnout (H4). Crucially, both pathways share the same cultural origin point but diverge in their downstream consequences. This dual-pathway architecture helps explain why cultural pressure may simultaneously drive performance-oriented effort via CSR and psychological cost via PD. Controlled regulation is inherently need-thwarting (Deci and Ryan, 2000), which creates conditions for affective depletion even when behavioural output is maintained.

A critical limitation of the existing literature is its near-exclusive reliance on cross-sectional designs, which cannot address three essential questions: (a) whether the proposed pathways reflect causal antecedence or shared-variance artefacts; (b) whether stable between-person differences in shame norm sensitivity confound the associations, a problem that random-intercept models are designed to address (Hamaker et al., 2015; Lucas, 2023); and (c) whether the pathways remain stable across time or respond to intervention. The few longitudinal studies in the SDT-sport literature have examined motivational change over competitive seasons or in response to coaching interventions (Ntoumanis et al., 2021), but none has simultaneously tracked cultural norm perception, motivational regulation, psychological distress, and objective athletic performance across multiple assessment waves in a Chinese sample. The present five-wave prospective design addresses this gap by enabling cross-lagged prospective associations and pre-post path comparisons that cross-sectional studies cannot provide.

A further gap concerns the cross-cultural validity of SDT-based sport psychology research. Although SDT claims universality of the autonomy, competence, and relatedness need triad, the motivational consequences of controlled versus autonomous regulation may be moderated by culturally embedded construals of the self (Tsang, 2025). In interdependent cultures, where the self is fundamentally constituted through group membership, introjected regulation, that is, training because one would feel ashamed not to train, may carry different phenomenological and functional properties than in individualist contexts (Ho, 1976). Chinese athletes face specific structural pressures, including the national sports system’s performance-outcome emphasis, Confucian obligations to coaches and families, and the dual academic-athletic demands of university sport (Woolf et al., 2024). This constellation is not captured by extant Western SDT frameworks. Research on Chinese athlete motivation remains relatively sparse, has relied predominantly on translated Western instruments without cultural adaptation, and has not systematically examined shame norms as a culturally specific antecedent. The present study addresses these shortcomings through the development and validation of the Sport Shame Norms Scale (SSNS), a six-item instrument designed to measure normative group-level shame perception rather than individual trait affect.

In addition to testing the baseline score-based model, the present study embeds the longitudinal assessment within an ACT-informed multimodal psychological skills training programme. The programme combined acceptance-based elements with cognitive-behavioural sport psychology components. Because shame norms are culturally embedded and not amenable to short-term individual change, the programme aimed not to eliminate normative pressure but to develop athletes’ psychological flexibility and coping repertoire: the capacity to remain in contact with shame-based thoughts without fusing with them, while also using structured attentional, self-talk, and pressure-management skills. By comparing T0 and T3 path coefficients, the study examines whether the score-based associations were attenuated after the programme, while avoiding causal claims because no no-intervention control group was included.

In summary, the present study tests the following *a priori* hypotheses in a sample of 345 competitive Chinese long-distance runners assessed across five waves:

*H1*: Shame norms will positively predict achievement pressure (SN→AP).

*H2a*: Achievement pressure will positively predict controlled self-regulation (AP→CSR).

*H2b*: Achievement pressure will not significantly predict autonomous self-regulation (AP→ASR; expected null).

*H3*: Controlled and autonomous self-regulation will jointly predict endurance performance (SR→EP).

*H4*: Achievement pressure and shame norms will positively predict psychological distress (AP, SN→PD).

**Figure 1:**
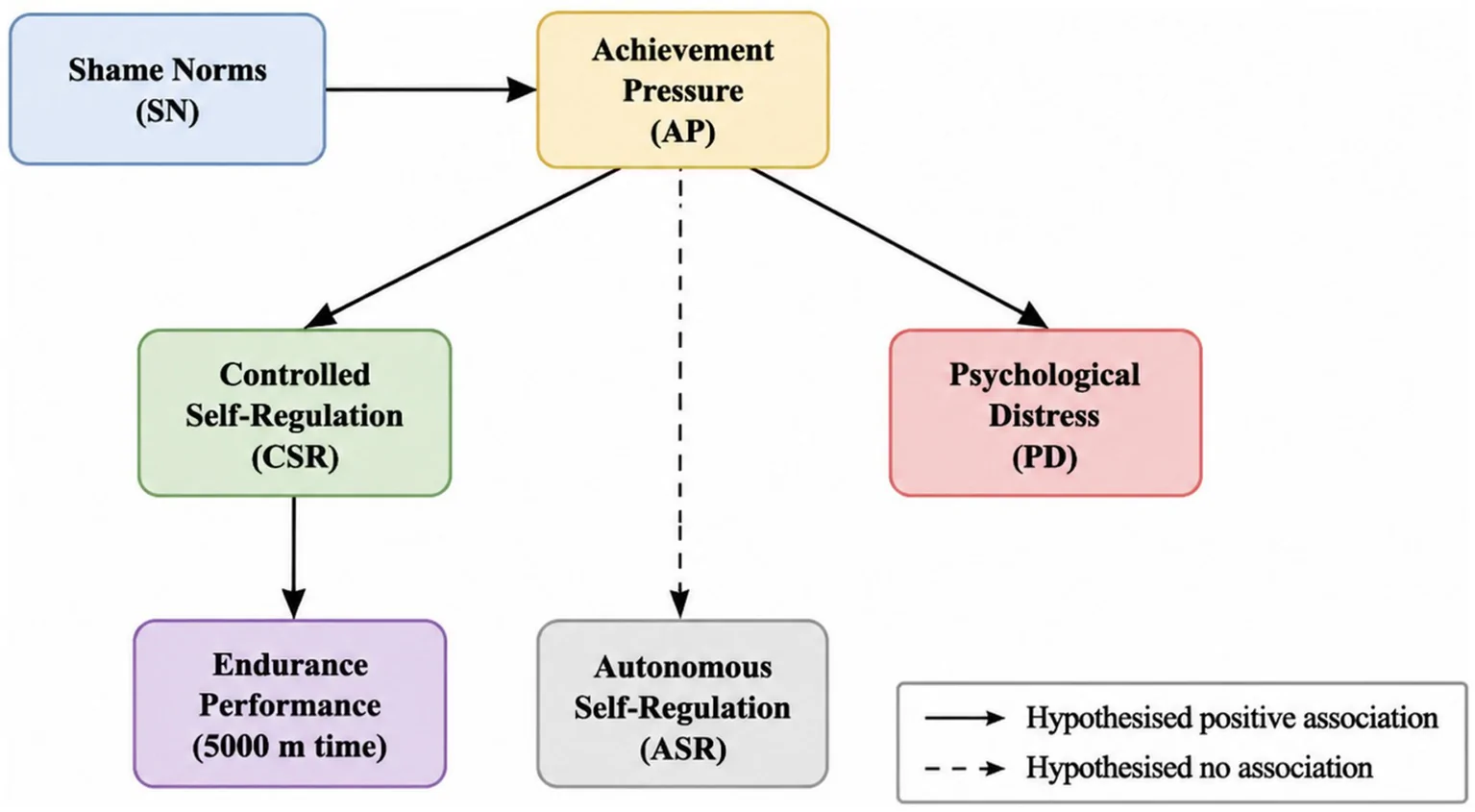
Conceptual dual-pathway model (hypothesised relationships).

The dual-pathway model was evaluated using baseline (T0) structural path analysis and five-wave cross-lagged associations (Figure 1). Temporal stability was assessed by comparing T0 and post-intervention (T3) path coefficients. Subgroup analyses further examined whether baseline shame norm levels moderated outcome magnitudes and intervention-related changes.

## 2 Materials and methods

### 2.1 Participants

**Figure 2:**
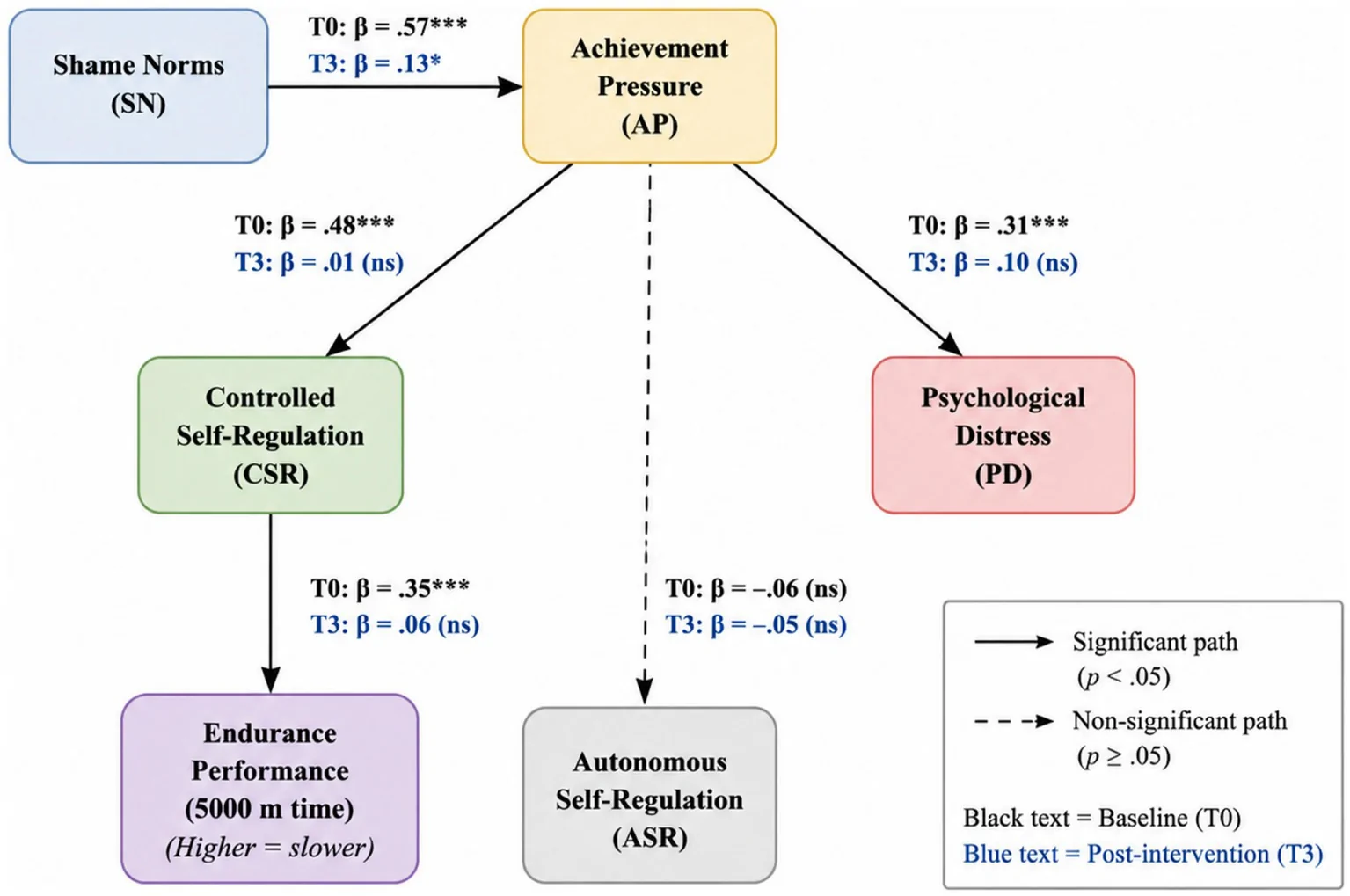
Empirical dual-pathway model at baseline (T0) and post-intervention (T3): standardized OLS path coefficients. Standardized ordinary least squares (OLS) regression coefficients are displayed for the hypothesised dual-pathway relationships. Black coefficients represent baseline estimates at T0 (pre-intervention), whereas blue coefficients represent post-intervention estimates at T3 (after the ACT-informed psychological skills training programme). Solid arrows indicate statistically significant paths (*p* < 0.05), and dashed arrows indicate non-significant paths (*p* ≥ 0.05). T0 estimates reflect the cultural motivational mechanism under naturalistic conditions, whereas T3 estimates indicate changes in path magnitude following the intervention. Endurance performance was operationalised as 5,000 m completion time, with higher values indicating slower performance.

A total of 388 competitive long-distance runners were initially recruited from five universities and four running clubs across three Chinese cities (Beijing, Chongqing, and Wuhan). Inclusion criteria were: (a) age 18–30 years; (b) minimum 12 months of systematic running training; (c) active competition in events ranging from 1,500 m to 10,000 m; (d) training frequency of at least three sessions per week; and (e) no current injury preventing participation in timed trials. Participants were excluded if they held national team status or were concurrently enrolled in another structured psychological intervention programme (Figure 2).

Sample size planning was based on established recommendations for longitudinal latent-variable research. Following Kline’s (2016) recommendation of a minimum sample size of approximately 200 to 250 participants for structural equation models involving multiple latent variables, and accounting for an anticipated attrition rate of approximately 11% across five measurement waves, an initial recruitment target of 388 participants was established. The final analytical strategy used latent structural equation modelling as the primary approach for testing the hypothesised dual-pathway framework while accounting for measurement error. Observed composite-score OLS regression models were additionally conducted as robustness analyses to examine whether the main conclusions remained consistent across alternative analytical approaches. During the study, 43 participants did not provide usable data across all five waves, attributable to sport injury, academic commitments, or other personal reasons. Specifically, 10 participants were lost at T1 due to sport injury (*n* = 6) or course scheduling conflicts (*n* = 4), 13 were lost at T2 due to sport injury (*n* = 8), examination preparation (*n* = 3), or internship commitments (*n* = 2), 13 were lost at T3 due to sport injury (*n* = 7), academic reasons (*n* = 4), or voluntary withdrawal for personal reasons (*n* = 2), and 7 were lost at T4 due to sport injury (*n* = 4), graduation and departure from university (*n* = 2), or loss to contact (*n* = 1). These 43 participants withdrew from the study entirely and are not part of the *N* = 345 analytic sample described below. This level of attrition is broadly comparable to that reported in similar longitudinal sport intervention research. However, baseline (T0) records for these 43 participants were not retained as part of the verified analytic data files; consequently, a formal completer-versus-dropout comparison on baseline characteristics could not be conducted, and this is acknowledged as a limitation in Section 4.6.

The final analytic sample comprised 345 participants who completed all five measurement waves, including 241 male athletes (69.9%) and 104 female athletes (30.1%), with a mean age of 21.04 years (SD = 1.73; range = 18 to 26; see Table 1). Completion was defined as having provided valid data for the core longitudinal variables across the study period; within this sample, 17 participants did not provide data specifically at the T3 wave due to scheduling conflicts that prevented participation in that assessment window. These 17 participants remained part of the N = 345 completer sample and contributed data at all other waves; they are retained in the repeated-measures analyses (Table 2), which accommodate wave-specific missingness, but are excluded from the T0–T3 paired comparisons (Table 3 note), which require a complete-paired subsample (*n* = 328). Primary analyses were conducted using this *N* = 345 complete-case analytic sample.

**Table 1:** Participant demographic and training characteristics (*N* = 345).

Characteristic	Category	*n*	%	M	SD	Range
Sex	Male	241	69.9			
	Female	104	30.1			
Age (years)				21.04	1.73	18–26
Academic year	Year 1	82	23.8			
	Year 2	96	27.8			
	Year 3	88	25.5			
	Year 4+	79	22.9			
Competitive level	School/intramural	74	21.4			
	University varsity	171	49.6			
	City/provincial	79	22.9			
	National club	21	6.1			
Training experience (yrs)				5.0	2.2	1–11
Weekly sessions				4.9	0.9	3–7
Weekly distance (km)				61.0	15.2	26–105
Personal best 5,000 m				17:59	98 s	14:49–22:15

*N* = 345. *M*, mean; SD, standard deviation.

**Table 2:** Repeated-measures ANOVA: means, SDs, and change statistics across T0–T4 (*N* = 345).

Variable	T0 M (SD)	T1 M (SD)	T2 M (SD)	T3 M (SD)	T4 M (SD)	*F*	*df*	*p*	*η* ^2^ *p*	d (T0–T3)	Trend
Shame norms: (SN)	3.79 (0.68)	3.76 (0.67)	3.68 (0.65)	3.55 (0.63)	3.58 (0.64)	15.74	3.2, 1039.2	<0.001	0.05	−0.31	Linear ↓
Achievement pressure: (AP)	3.59 (0.70)	3.57 (0.68)	3.47 (0.66)	3.36 (0.63)	3.42 (0.62)	12.41	3.0, 995.6	<0.001	0.04	−0.27	Linear ↓
Controlled SR: (CSR)	3.48 (0.72)	3.57 (0.71)	3.72 (0.69)	3.85 (0.63)	3.83 (0.64)	46.73	2.4, 771.5	<0.001	0.13	0.50	Linear ↑
Autonomous SR: (ASR)	3.84 (0.63)	3.87 (0.59)	3.89 (0.56)	3.89 (0.55)	3.89 (0.56)	3.67	2.1, 671.5	0.02	0.01	0.13	Linear ↑
Psychological distress: (PD)	2.71 (0.80)	2.64 (0.78)	2.52 (0.75)	2.34 (0.71)	2.36 (0.73)	29.54	3.1, 1009.0	<0.001	0.08	−0.43	Linear ↓
Perceived exertion: (PE)	6.88 (1.09)	6.79 (1.07)	6.63 (1.03)	6.45 (0.99)	6.49 (1.02)	17.60	3.1, 1013.7	<0.001	0.05	−0.32	Linear ↓
Burnout index: (BI)	2.37 (0.75)	2.34 (0.74)	2.15 (0.66)	2.06 (0.63)	2.12 (0.67)	25.46	3.2, 1053.8	<0.001	0.07	−0.39	Linear ↓
Social support: (SS)	3.94 (0.70)	3.93 (0.70)	3.97 (0.70)	4.01 (0.70)	4.01 (0.70)	1.40	3.3, 1069.6	0.24	0.00	0.09	Linear ↑
5,000 m time—sec: (EP)	1071.00 (96.51)	1058.81 (93.92)	1044.34 (88.93)	1022.57 (84.66)	1025.15 (87.21)	34.95	2.8, 899.3	<0.001	0.10	−0.46	Linear ↓

Descriptive M(SD) values are based on wave-specific valid observations (T0 = 345, T1 = 338, T2 = 332, T3 = 328, T4 = 333). Repeated-measures ANOVA was conducted on the fixed complete repeated-measures subsample (*N* = 328) using pingouin.rm_anova (Vallat, 2018) with Greenhouse–Geisser correction where applicable. *η*^2^*p* = partial eta-squared. Cohen’s d is based on the fixed T0–T3 paired subsample (*N* = 328). CAUTION: Single-arm design; changes cannot be causally attributed to intervention.

**Table 3:** Paired comparison T0 vs. T3 (post-intervention): *T*-tests and effect sizes.

Variable	T0 M (SD)	T3 M (SD)	Δ M (T3 − T0)	*t*	*df*	*p* (two-tailed)	Cohen’s *d*	95% CI (*d*)
Shame norms (SN)	3.79 (0.69)	3.55 (0.63)	−0.24	5.57	327	<0.001	−0.31	−0.42, −0.20
Achievement pressure (AP)	3.58 (0.70)	3.36 (0.63)	−0.22	4.96	327	<0.001	−0.27	−0.38, −0.16
Controlled SR (CSR)	3.47 (0.72)	3.85 (0.63)	0.38	−9.06	327	<0.001	0.50	0.39, 0.61
Autonomous SR (ASR)	3.84 (0.62)	3.89 (0.55)	0.06	−2.35	327	0.01	0.13	0.02, 0.24
Psychological distress (PD)	2.72 (0.80)	2.34 (0.71)	−0.38	7.82	327	<0.001	−0.43	−0.55, −0.32
Perceived exertion (PE)	6.87 (1.10)	6.45 (0.99)	−0.42	5.87	327	<0.001	−0.32	−0.43, −0.21
Burnout index (BI)	2.37 (0.75)	2.06 (0.63)	−0.31	7.03	327	<0.001	−0.39	−0.50, −0.28
Social support (SS)	3.94 (0.71)	4.01 (0.70)	0.08	−1.63	327	0.10	0.09	−0.02, 0.20
5,000 m time—sec (EP)	1069.52 (96.65)	1022.57 (84.66)	−46.95	8.26	327	<0.001	−0.46	−0.57, −0.34

Paired *t*-tests (two-tailed), conducted using scipy.stats.ttest_rel. Cohen’s d was calculated as the mean difference divided by the standard deviation of the differences. Paired t-tests were restricted to the 328 participants with complete T0 and T3 data across all variables; the reduced N relative to the full analytic sample (*N* = 345) reflects this fixed T0–T3 paired-subsample definition rather than variable-specific missingness. This is why the degrees of freedom (*df* = 327) are identical across all nine variables. The small difference between the 5,000 m baseline value reported in Tables 2, 3 reflects this fixed-subsample basis (*N* = 345 vs. *n* = 328) rather than a different participant sample or a different performance variable.

### 2.2 Research design

A longitudinal single-arm intervention design with five measurement waves was employed. Waves were administered at: T0 (baseline, pre-intervention), T1 (Week 2, early intervention), T2 (Week 4, mid-intervention), T3 (Weeks 6–8, post-intervention, primary endpoint), and T4 (Week 12, four-week follow-up). At each wave, participants completed a self-report questionnaire battery and a standardised 5,000 m time trial on separate days within a 72-h window to avoid acute fatigue contaminating psychological self-reports.

The absence of a no-intervention control group is an acknowledged limitation of this design, arising from ethical constraints withholding a structured psychological skills programme from competitive athletes during an active training season—and from the practical unavailability of a matched comparison cohort. Accordingly, observed pre-to-post changes are reported descriptively and do not support causal attribution to the intervention. The primary inferential value of the longitudinal design lies in (a) the T0 baseline path analysis, which examines the cultural mechanism under naturalistic conditions, and (b) the cross-lagged prospective associations, which provide time-ordered evidence for the proposed directional relationships.

### 2.3 Intervention

The psychological skills training (PST) programme was ACT-informed and multimodal rather than pure ACT. It was grounded in Acceptance and Commitment Training (Hayes et al., 2012) and cognitive-behavioural sport psychology (CBSP; Thelwell et al., 2010). The ACT-informed components targeted psychological flexibility and cognitive defusion from shame-based self-narratives, whereas the CBSP components targeted attentional focus, instructional self-talk, and pressure rehearsal. This revised terminology is used throughout to avoid overstating the theoretical specificity of the active ingredients.

The programme comprised six to eight weekly 60-min group sessions supplemented by daily 10–15 min individual practice, organised into five sequential modules: (1) psychoeducation (Weeks 1–2): normalising achievement pressure in Chinese sport culture and introducing the distinction between controlled and autonomous motivation; (2) cognitive defusion (Weeks 2–3): techniques for observing shame-related thoughts without literal fusion or behavioural avoidance; (3) attention regulation and self-talk (Weeks 3–5): instructional self-talk linked to pacing strategy and attentional focus shift training; (4) goal setting and committed action (Weeks 5–6): values-based process goals replacing social-comparison performance goals; and (5) stress inoculation (Weeks 6–8): simulated high-pressure training scenarios with debriefing.

Sessions were facilitated by two registered sport psychologists, both holding ACT and applied sport psychology training with a minimum of 5 years of applied experience. Programme fidelity was monitored via session checklists completed after each group session; mean fidelity was 94.2% (SD = 3.8%) across all sessions and facilitators.

### 2.4 Measures

All questionnaire items were administered in Mandarin Chinese. English-language scales were translated using Brislin’s (1980) back-translation protocol: (1) forward translation by a bilingual sport psychologist; (2) independent back-translation by a second bilingual translator blind to the original wording; (3) expert panel review (*n* = 5) to resolve discrepancies; and (4) cognitive pilot testing with 32 athletes not included in the main sample, with item clarity rated ≥ 4.2 out of 5 for all items.

#### 2.4.1 Shame norms

Shame norms were assessed using the Sport Shame Norms Scale (SSNS), a six-item measure developed for this study to capture perceived group-level standards linking athletic failure to embarrassment, loss of face, and collective disgrace. The scale was conceptually informed by research on face and shame in social-evaluative contexts (Ho, 1976; Tangney and Dearing, 2002; Tangney et al., 2007), but it was not treated as a direct transplantation of an organizational face-concern scale. This clarification is important because shame norms in competitive training groups refer to perceived team or peer standards rather than dyadic workplace concern about a supervisor’s evaluation. Items were reviewed by 2 sport psychology experts and pilot tested during the translation and adaptation procedure described above. Items are rated on a 5-point Likert scale (1 = strongly disagree to 5 = strongly agree); higher scores reflect stronger perceived shame norms. Cronbach’s *α* = 0.85 in the present sample.

#### 2.4.2 Achievement pressure

Achievement pressure was measured using the seven-item Performance Climate subscale of the Perceived Motivational Climate in Sport Questionnaire 2 (PMCSQ-2; Newton et al., 2000), rated on a 5-point Likert scale (1 = strongly disagree to 5 = strongly agree). This subscale captures the extent to which athletes perceive their motivational environment as emphasising normative performance and punishing mistakes. Cronbach’s α = 0.88.

#### 2.4.3 Behavioural regulation

Motivational self-regulation was assessed using the Behavioural Regulation in Exercise Questionnaire–3 (BREQ-3; Luo et al., 2022), a self-determination theory-based measure that captures external, introjected, identified, and intrinsic forms of regulation. Controlled self-regulation (CSR) was computed as the mean of the external regulation (four items; e.g., “I exercise because other people say I should”) and introjected regulation (four items; e.g., “I would feel ashamed if I did not make the effort to exercise”) subscales—the two regulation types driven by external contingencies and internalised pressure, respectively. Autonomous self-regulation (ASR) was computed as the mean of the identified regulation (four items) and intrinsic regulation (four items) subscales. Items are rated on a 4-point scale (1 = not true for me to 4 = very true for me). This operationalisation directly tests the theoretical prediction that shame-driven pressure selectively activates controlled rather than autonomous regulation (H2a vs. H2b). Cronbach’s *α* = 0.87 (CSR) and 0.93 (ASR). Because BREQ-3 is exercise-framed, its use with competitive endurance athletes is interpreted cautiously. Competitive runners engaged in structured training may interpret exercise wording differently from recreational exercisers. Future work should compare these results with sport-specific measures such as the Behavioral Regulation in Sport Questionnaire and test whether item wording introduces construct misrepresentation in competitive samples.

#### 2.4.4 Psychological distress

Psychological distress was assessed using the Kessler Psychological Distress Scale (K10; Kessler et al., 2002), a 10-item measure of anxiety and depressive symptoms experienced over the past 4 weeks. Items are rated on a 5-point scale (1 = none of the time to 5 = all of the time). For consistency with the other scale-score analyses and tables, the present analyses used item-mean scores on the original 1–5 response metric rather than summed 10–50 scores; higher mean scores indicate greater distress. Cronbach’s *α* was 0.86 in the present sample.

#### 2.4.5 Perceived exertion, burnout, and social support

Perceived exertion (PE) was assessed post-time-trial using five items adapted from Borg’s (1982) Rating of Perceived Exertion scale and Marcora’s (2009) effort perception framework, rated on a 1–10 numeric scale (α = 0.84). Athlete burnout was measured using the exhaustion (five items) and devaluation (four items) subscales of the Athlete Burnout Questionnaire (ABQ; Raedeke and Smith, 2001), rated on a 5-point scale (*α* = 0.84). Social support was assessed with the six-item sport social support scale (SSSS; Freeman et al., 2011; *α* = 0.80). It was evaluated as a possible marker variable during common method bias checks but was not retained as a marker correction because it was theoretically and empirically related to the substantive constructs (see Section 2.5).

#### 2.4.6 Endurance performance

Objective endurance performance was assessed via a standardised 5,000 m time trial conducted on a 400 m outdoor synthetic track (IAAF Class 2 certified). A detailed standardisation protocol governed each wave, covering: testing window (08:00–10:00 local time), environmental exclusion criteria (temperature < 5 °C or > 28 °C, relative humidity > 80%), pre-test nutrition (no food 2 h prior; 500 mL water 1 h prior), warm-up (10-min group jog plus four × 100 m strides), and training load restrictions (rest day at T − 2, light jog only at T − 1). Timing was recorded using a fully automatic timing system (FAT) with manual backup. Performance is expressed in seconds (lower values = faster performance). Nine data points across all waves involved protocol deviations (<0.5% of observations); sensitivity analyses including and excluding these cases produced identical conclusions.

### 2.5 Common method bias controls

Given that all psychological variables were collected via self-report questionnaire, procedural and statistical diagnostics for common method bias (CMB; Podsakoff et al., 2003) were implemented. Procedurally, psychological distress and burnout were administered 48 h apart, two reverse-scored distractor items were embedded in the shame norms scale, distress items were framed as a general wellbeing check rather than labelled as a psychological symptom measure, and perceived exertion was completed immediately post-time-trial in a physically separate context from the questionnaire battery. Statistically, Harman’s single-factor diagnostic indicated that the first unrotated factor accounted for 22.1% of item variance, below the conventional 50% heuristic. A marker-variable correction was not retained because social support was theoretically and empirically related to the substantive constructs and was therefore not orthogonal. The CMB evidence should be interpreted as diagnostic rather than as eliminating common method bias.

### 2.6 Data analysis

All analyses were conducted using the accompanying reproducible Python workflow and standard statistical packages. Descriptive statistics, Cronbach’s alpha, zero-order Pearson correlations, CFA-based measurement evaluation, composite reliability (CR), average variance extracted (AVE), HTMT ratios, longitudinal measurement analyses, repeated-measures summaries, Welch subgroup tests, cross-lagged partial correlations, latent structural equation models, and observed-score OLS robustness analyses were computed from the item-level datasets. A baseline confirmatory factor analysis was first conducted to evaluate the eight-factor measurement structure. Longitudinal measurement invariance across the five assessment waves (T0 to T4) was subsequently examined through sequential configural, metric, scalar, and strict invariance models. Changes in CFI and RMSEA were evaluated across nested models (Cheung and Rensvold, 2002), and latent mean differences were estimated after establishing scalar invariance. Detailed measurement invariance results are reported. The hypothesised dual-pathway framework was primarily evaluated using latent structural equation models to account for measurement error and estimate relationships among theoretical constructs. Wave-specific latent SEM models were estimated at T0 and T3 to examine whether the proposed structural relationships were consistent across assessment points. Observed-score OLS regression analyses were subsequently conducted as robustness analyses to examine whether the main conclusions remained consistent when using composite-score estimates. All variables were z-standardised before OLS estimation to allow comparable interpretation of coefficients across equations. Because observed-score models do not explicitly account for measurement error, OLS findings were interpreted as robustness estimates rather than replacements for latent structural analyses.

Repeated-measures analyses were used to characterise longitudinal changes descriptively. Because the study employed a single-arm design without a no-intervention control group, pre to post changes were interpreted as temporal changes rather than causal intervention effects. Potential confounding by training experience, weekly running distance, and competitive level was not formally tested in the present analyses and is noted as a limitation in Section 4.6. Because baseline (T0) records for the 43 participants who withdrew before completing all five waves were not retained in the verified analytic data files, a formal comparison between completers and non-completers on baseline characteristics could not be conducted; this is likewise acknowledged as a limitation in Section 4.6. To examine prospective directional associations across the five waves, cross-lagged partial correlations were calculated between adjacent waves while controlling for prior-wave levels of the outcome variables. This approach provides time-ordered observed-score evidence while accounting for autoregressive stability; however, it does not separate stable between-person differences from within-person fluctuations. A full random-intercept cross-lagged panel model (RI-CLPM) was not estimated; therefore, these cross-lagged associations were interpreted cautiously as mixed between-person and within-person associations rather than definitive within-person causal effects. Finally, subgroup analyses examined whether baseline shame norm levels differentiated outcomes. Participants were divided at the T0 median (Mdn = 3.88) into High-SN (*n* = 177) and Low-SN (*n* = 168) groups. Independent-samples Welch t-tests were conducted with Welch-Satterthwaite degrees of freedom recalculated according to subgroup variances. Cohen’s d and 95% confidence intervals were reported as effect-size indicators. Bayes factors (BF10; Rouder et al., 2009) were reported only for subgroup comparisons and were not interpreted as evidence confirming null structural paths.

## 3 Results

### 3.1 Descriptive statistics and scale reliability

As shown in Table 4, the baseline correlation pattern was broadly consistent with the proposed dual pathway framework. Shame norms and achievement pressure were strongly positively associated, and both were positively related to controlled self regulation and psychological distress. In contrast, achievement pressure was not significantly associated with autonomous self regulation, supporting the prediction that culturally driven pressure selectively activates controlled motivation. The endurance outcome requires careful interpretation. Because 5,000 m completion time was coded in seconds, with higher values indicating slower performance, positive correlations with shame norms, achievement pressure, and both forms of self regulation reflect slower performance. The negative associations of psychological distress and burnout with completion time are counterintuitive and suggest that objective performance is shaped by a more complex combination of motivational, physiological, and training history factors than a simple linear relation between wellbeing and performance would imply (Table 4).

**Table 4:** Descriptive statistics and zero order correlations at baseline (T0, *N* = 345).

Variable	M	SD	*α*	1	2	3	4	5	6	7	8	9
1. Shame norms (SN)	3.79	0.68	0.85	—								
2. Achievement pressure (AP)	3.59	0.70	0.88	0.57***	—							
3. Controlled SR (CSR)	3.48	0.72	0.87	0.48***	0.48***	—						
4. Autonomous SR (ASR)	3.84	0.63	0.93	−0.13*	−0.06	−0.04	—					
5. Psychological distress (PD)	2.71	0.80	0.86	0.48***	0.48***	0.19***	−0.31***	—				
Perceived exertion (PE)	6.88	1.09	0.84	0.40***	0.40***	0.26***	−0.05	0.49***	—			
7. Burnout index (BI)	2.37	0.75	0.84	0.38***	0.43***	0.22***	−0.22***	0.60***	0.40***	—		
8. Social support (SS)	3.94	0.70	0.80	−0.29***	−0.29***	−0.08	0.16**	−0.49***	−0.20***	−0.44***	—	
9. 5,000 m time—sec (EP)	1071.0	96.5	—	0.29***	0.27***	0.34***	0.25***	−0.23***	0.43***	−0.20***	0.19***	—

*N* = 345. *α* = Cronbach’s alpha. 5000 m time in seconds (lower = faster). *p < 0.05; **p < 0.01; ***p < 0.001 (two-tailed). Controlled SR = introjected + external regulation (BREQ-3); Autonomous SR = identified + intrinsic regulation (BREQ-3).

Table 5 reports measurement-quality and common-method diagnostics. The baseline eight-factor CFA showed acceptable fit, *χ*^2^(1624) = 1815.21, CFI = 0.977, TLI = 0.975, RMSEA = 0.019 (Hu and Bentler, 1999). CR values ranged from 0.749 to 0.926. AVE values were strongest for ASR and PE and weaker for PD, BI, and social support, indicating that some adapted measures require further validation. HTMT values ranged from low levels to a maximum of 0.706, below the 0.85 threshold (Henseler et al., 2015), supporting discriminant validity among the focal constructs. The one-factor CFA fit substantially worse than the eight-factor model (CFI = 0.470, TLI = 0.451, RMSEA = 0.087), and the Harman diagnostic suggested that the first unrotated factor accounted for 22.1% of variance, so no dominant single factor was observed. These diagnostics support the use of the observed scale scores while also highlighting the importance of interpreting observed-score estimates cautiously because composite-score OLS models were conducted only as robustness analyses alongside the primary latent structural models.

**Table 5:** Measurement quality, construct validity, and common-method diagnostics.

Construct/diagnostic	Items/statistic	Reliability (*α*)	Factor loading range	CR	AVE	Interpretation
Shame norms	6	0.847	0.631–0.764	0.847	0.482	Preliminary acceptable; AVE interpreted cautiously
Achievement pressure	7	0.649	0.706–0.760	0.786	0.507	Acceptable
Controlled self-regulation	8	0.872	0.620–0.720	0.872	0.462	Preliminary acceptable; AVE interpreted cautiously
Autonomous self-regulation	8	0.926	0.747–0.800	0.926	0.610	Acceptable
Psychological distress	10	0.857	0.568–0.672	0.857	0.375	Preliminary acceptable; AVE interpreted cautiously
Perceived exertion	5	0.837	0.673–0.739	0.837	0.507	Acceptable
Burnout indicators	9	0.675	0.642–0.668	0.749	0.364	Preliminary acceptable; AVE interpreted cautiously
Social support	6	0.796	0.557–0.677	0.798	0.397	Preliminary acceptable; AVE interpreted cautiously
Eight-factor baseline CFA	59 items	1815.21	1,624	0.977	0.975/0.019	Acceptable baseline measurement structure
One-factor CFA diagnostic	59 items	5992.28	1,652	0.470	0.451/0.087	Substantially weaker fit; diagnostic only
HTMT discriminant validity	28 construct pairs	Max HTMT = 0.706	Criterion <0.85	—	—	Supports discriminant validity among observed constructs
Harman single-factor diagnostic	59 items	First factor = 22.1%	—	—	—	No dominant single factor; common method bias not eliminated

CFA fit statistics are reported as *χ*^2^, *df*, CFI, TLI, and RMSEA. CR = composite reliability; AVE = average variance extracted; HTMT = heterotrait–monotrait ratio.

### 3.2 Latent structural equation model testing of the dual-pathway framework

To provide the primary test of the hypothesized dual-pathway framework while accounting for measurement error, latent structural equation models were estimated. The latent SEM results supported the proposed theoretical structure and showed a pattern consistent with the hypothesized relationships. The model fit indices indicated an acceptable representation of the measurement and structural model at both assessment points (Table 6). The observed-score OLS analyses were subsequently conducted as robustness checks to examine whether the main conclusions remained consistent across analytical approaches.

**Table 6:** Latent structural equation model estimates and model fit for the dual-pathway framework.

Outcome	Predictor	Hypothesis	T0 *β*/CFI	T0 *p*/TLI	T3 *β*/RMSEA	T3 *p*/SRMR
Panel A. Standardized latent SEM path estimates						
AP	SN	H1: SN → AP	0.696	<0.001	0.154	0.02
CSR	AP	H2a: AP → CSR	0.568	<0.001	−0.012	0.853
ASR	AP	H2b: AP → ASR	−0.077	0.194	−0.065	0.29
EP_sec	CSR	H3: CSR → EP	0.375	<0.001	0.054	0.355
EP_sec	ASR	H3: ASR → EP	0.26	<0.001	0.082	0.154
PD	AP	H4: AP → PD	0.246	0.001	0.111	0.089
PD	SN	Supplementary: SN → PD	0.255	0.002	0.063	0.34
PD	SS	Covariate: SS → PD	−0.422	<0.001	−0.112	0.093

Panel B. model fit indices	Wave	*χ*^2^(df); *χ*^2^/df	CFI	TLI	RMSEA	SRMR
	T0	N = 345; *χ*^2^(980) = 1221.55; *χ*^2^/df = 1.25	0.963	0.961	0.027	0.071
	T3	N = 328; *χ*^2^(980) = 1012.93; *χ*^2^/df = 1.03	0.994	0.994	0.010	0.047

Latent structural equation models were estimated for the hypothesised dual-pathway framework. Standardized estimates are reported. EP_sec was entered as an observed performance outcome because it was objectively assessed through the 5,000 m time trial. Wave-specific latent SEM models were estimated separately at T0 and T3 and should not be interpreted as longitudinal causal models.

### 3.3 Longitudinal changes across the intervention period (T0–T4)

Table 2 shows clear longitudinal trends across the five measurement waves. Shame norms and achievement pressure declined over time, while both controlled and autonomous self regulation increased. Psychological distress, perceived exertion, and burnout all showed consistent decreases, and 5,000 m completion time declined, indicating faster running performance at later waves. Social support remained relatively stable. These patterns suggest that the study period was associated with broad improvements in both psychological functioning and objective endurance outcomes, particularly between T1 and T3. However, because no control group was included, these changes should be interpreted descriptively rather than causally (see Table 2).

The paired comparisons reported in Table 3 indicate statistically significant improvements from T0 to T3 across most key variables. Shame norms, achievement pressure, psychological distress, perceived exertion, burnout, and 5,000 m completion time all decreased, while both forms of self regulation increased. The effect sizes suggest that the largest changes occurred in controlled self regulation and psychological distress. These findings provide evidence of meaningful within sample change over the intervention window. However, given that the analyses were conducted on complete cases for the relevant time points, and in the absence of a comparison group, the results should be interpreted as indicative of temporal change rather than as evidence of causal intervention effects (see Table 2).

### 3.4 Subgroup analyses: high vs. low shame norm athletes

The subgroup results presented in Table 7 show a clear baseline disadvantage for athletes high in shame norms, who reported greater achievement pressure, controlled self regulation, psychological distress, perceived exertion, and burnout, along with slower 5,000 m completion times. Autonomous self regulation did not differ significantly between groups, indicating that shame norms selectively elevated controlled rather than autonomous motivation. Over time, the High SN group showed larger reductions in psychosocial burden, particularly in achievement pressure, distress, and exertion. For endurance performance, the High SN group showed a numerically larger reduction in completion time, but the effect size was small and the Bayesian evidence was inconclusive. These findings indicate strong evidence for differential psychosocial responsiveness, but only weak evidence for corresponding differences in short term performance gain (see Table 7).

**Table 7:** High-SN vs. low-SN subgroup comparisons at T0, T3, and T0–T3 change.

Variable	Wave	High SN M (SD)	Low SN M (SD)	*t*	*df*	*p*	Cohen’s *d*	95% CI (*d*)	BF₁₀
Shame norms (SN)	T0	4.34 (0.30)	3.21 (0.44)	27.42	294.6	<0.001	2.98	2.67, 3.29	>1,000
	T3	3.75 (0.57)	3.34 (0.63)	6.08	315.5	<0.001	0.67	0.45, 0.90	>1,000
	*Δ* (T0–T3)	−0.60 (0.60)	0.15 (0.77)	−9.73	297.4	<0.001	−1.08	−1.32, −0.85	>1,000
Achievement pressure (AP)	T0	3.90 (0.58)	3.25 (0.65)	9.82	333.5	<0.001	1.06	0.84, 1.29	>1,000
	T3	3.45 (0.61)	3.27 (0.64)	2.52	315.8	0.01	0.28	0.06, 0.50	2.5
	Δ (T0–T3)	−0.46 (0.71)	0.04 (0.83)	−5.90	309.5	<0.001	−0.66	−0.88, −0.43	>1,000
Controlled SR (CSR)	T0	3.76 (0.42)	3.18 (0.84)	8.05	252.1	<0.001	0.88	0.66, 1.10	>1,000
	T3	3.94 (0.40)	3.76 (0.80)	2.56	241.7	0.01	0.29	0.07, 0.51	2.8
	Δ (T0–T3)	0.18 (0.51)	0.59 (0.91)	−4.88	265.6	<0.001	−0.55	−0.77, −0.33	>1,000
Autonomous SR (ASR)	T0	3.78 (0.72)	3.90 (0.51)	−1.71	323.0	0.08	−0.18	−0.39, 0.03	0.5
	T3	3.88 (0.56)	3.91 (0.53)	−0.34	326.0	0.73	−0.04	−0.25, 0.18	0.1
	Δ (T0–T3)	0.09 (0.57)	0.02 (0.23)	1.65	255.5	0.10	0.18	−0.04, 0.40	0.5
Psychological distress (PD)	T0	3.04 (0.77)	2.37 (0.68)	8.58	341.4	<0.001	0.92	0.70, 1.14	>1,000
	T3	2.42 (0.72)	2.25 (0.69)	2.23	325.7	0.02	0.25	0.03, 0.46	1.3
	Δ (T0–T3)	−0.62 (0.87)	−0.12 (0.80)	−5.45	326.0	<0.001	−0.60	−0.82, −0.38	>1,000
Perceived exertion (PE)	T0	7.22 (1.00)	6.51 (1.07)	6.33	337.8	<0.001	0.68	0.47, 0.90	>1,000
	T3	6.49 (0.99)	6.40 (0.99)	0.80	323.7	0.42	0.09	−0.13, 0.31	0.2
	Δ (T0–T3)	−0.74 (1.29)	−0.08 (1.21)	−4.77	325.9	<0.001	−0.53	−0.75, −0.31	>1,000
Burnout index (BI)	T0	2.59 (0.76)	2.14 (0.66)	5.92	341.9	<0.001	0.64	0.42, 0.85	>1,000
	T3	2.11 (0.64)	2.01 (0.61)	1.44	325.9	0.15	0.16	−0.06, 0.38	0.3
	Δ (T0–T3)	−0.48 (0.82)	−0.14 (0.76)	−3.88	325.0	<0.001	−0.43	−0.65, −0.21	144.3
Social support (SS)	T0	3.79 (0.74)	4.10 (0.63)	−4.30	339.1	<0.001	−0.46	−0.67, −0.25	700.1
	T3	4.00 (0.70)	4.04 (0.70)	−0.53	323.8	0.59	−0.06	−0.28, 0.16	0.1
	Δ (T0–T3)	0.21 (0.84)	−0.06 (0.80)	2.97	325.6	0.00	0.33	0.11, 0.55	8.0
5,000 m time—sec (EP)	T0	1090.79 (96.53)	1050.14 (92.27)	4.00	343.0	<0.001	0.43	0.22, 0.64	224.7
	T3	1034.01 (78.47)	1010.27 (89.48)	2.55	313.1	0.01	0.28	0.07, 0.50	2.7
	Δ (T0–T3)	−57.76 (103.31)	−35.31 (101.45)	−1.99	325.0	0.04	−0.22	−0.44, −0.00	0.8

Median split on T0 SN_mean (Mdn = 3.88): high SN *n* = 177, low SN *n* = 168. T3 and Δ (T0–T3) comparisons were restricted to participants with complete data at both T0 and T3 (High-SN *n* = 170, Low-SN *n* = 158, total *n* = 328), consistent with the complete-paired subsample used in Tables 2, 3; the 17 participants missing at T3 (see Section 2.1) are excluded from these rows but retained in the T0-only comparisons (High-SN *n* = 177, Low-SN *n* = 168). Independent-samples Welch t-tests are reported with Welch-Satterthwaite degrees of freedom recalculated separately for each row from the observed subgroup variances and the corresponding group sizes noted above. Cohen’s *d* = (M_High − M_Low)/SD_pooled. BF10 = Bayes factor (Cauchy prior *r* = 0.707; JZS approximation). BF values are descriptive for subgroup comparisons and are not used to confirm null structural paths.

### 3.5 Prospective cross-lagged associations

The cross lagged partial correlations reported in Table 8 provide limited time ordered support for the proposed directional relationships. At the earliest interval, shame norms predicted later achievement pressure, which in turn predicted both controlled self regulation and psychological distress, and controlled self regulation predicted subsequent endurance outcome. However, these forward associations weakened at later intervals, and several reverse paths were also significant at early transitions. This pattern indicates that the hypothesised sequence was most evident at the T1 to T2 interval, but that directional exclusivity was not observed. The findings therefore support the proposed model in a limited sense, while also suggesting that within person dynamics are less stable across time than the baseline structure might imply (see Table 8).

**Table 8:** Cross-lagged partial correlations: prospective associations between adjacent waves (controlling for prior-wave outcome level).

Predictor (wave *t*)	Outcome (wave *t* + 1)	Waves	*r*	*p*	95% CI	*n*	Interpretation
AP	CSR	T1 → T2	0.15	0.00	0.05, 0.26	332	✓ prospective association
	CSR	T2 → T3	0.04	0.41	−0.06, 0.15	328	—
	CSR	T3 → T4	0.00	0.97	−0.11, 0.11	328	—
CSR	EP	T1 → T2	0.11	0.04	0.00, 0.21	332	✓ prospective association
	EP	T2 → T3	0.07	0.21	−0.04, 0.18	328	—
	EP	T3 → T4	0.00	0.98	−0.11, 0.11	328	—
AP	PD	T1 → T2	0.20	< 0.001	0.10, 0.30	332	✓ prospective association
	PD	T2 → T3	0.16	0.00	0.05, 0.26	328	✓ prospective association
	PD	T3 → T4	0.07	0.22	−0.04, 0.17	328	—
SN	AP	T1 → T2	0.16	0.00	0.05, 0.26	332	✓ prospective association
	AP	T2 → T3	0.08	0.14	−0.03, 0.19	328	—
	AP	T3 → T4	0.07	0.19	−0.04, 0.18	328	—
CSR	AP (reverse)	T1 → T2	0.15	0.00	0.04, 0.25	332	—
	AP (reverse)	T2 → T3	−0.04	0.47	−0.15, 0.07	328	NS—reverse not supported
	AP (reverse)	T3 → T4	−0.03	0.61	−0.14, 0.08	328	NS—reverse not supported
EP	CSR (reverse)	T1 → T2	0.14	0.01	0.03, 0.24	332	—
	CSR (reverse)	T2 → T3	0.11	0.04	0.00, 0.22	328	—
	CSR (reverse)	T3 → T4	0.01	0.91	−0.10, 0.11	328	NS—reverse not supported
PD	AP (reverse)	T1 → T2	0.15	0.00	0.04, 0.25	332	—
	AP (reverse)	T2 → T3	0.18	< 0.001	0.08, 0.29	328	—
	AP (reverse)	T3 → T4	0.06	0.24	−0.04, 0.17	328	NS—reverse not supported

Cross-lagged partial correlations controlling for prior level of the outcome variable. Green = theory-predicted association confirmed; amber = unexpected reverse association. These are observational associations, not causal estimates.

### 3.6 Observed-score OLS robustness analyses

To evaluate the robustness and transparency of the primary latent SEM findings, observed-score OLS regression analyses were additionally conducted using composite scores. Although latent SEM provides the primary test of the hypothesised dual-pathway framework by accounting for measurement error, observed-score analyses allow direct comparison with the score-based associations commonly reported in applied sport psychology research and provide an accessible examination of whether the main theoretical patterns remain consistent across analytical approaches. The OLS models were estimated separately at baseline (T0) and post-intervention (T3), with all variables standardised before analysis. As shown in Table 9, the observed-score regression results demonstrated a pattern broadly consistent with the latent SEM estimates, supporting the stability of the hypothesised associations while also highlighting attenuation of several pathways at T3. Given the single-arm design and the absence of a control group, differences between T0 and T3 coefficients are interpreted as changes in association magnitude rather than causal intervention effects.

**Table 9:** Standardised OLS regression: hypothesised paths at baseline (T0) and post-intervention (T3).

Outcome	Predictor	Hypothesis	T0 *β*	T0 p	T3 *β*	T3 *p*	95% CI T0	95% CI T3	*R*^2^ T0	*R*^2^ T3	Note
AP_mean—H1: SN → AP											
AP_mean	SN_mean	H1: SN → AP	0.57	<0.001	0.13	0.02	0.49, 0.66	0.02, 0.24	0.33	0.02	H supported (T3)
CSR_mean—H2a: AP → CSR											
CSR_mean	AP_mean	H2a: AP → CSR	0.48	<0.001	0.01	0.86	0.38, 0.57	−0.10, 0.12	0.23	0.00	—
ASR_mean—H2b: AP → ASR (expect ns)											
ASR_mean	AP_mean	H2b: AP → ASR (expect ns)	−0.06	0.27	−0.05	0.38	−0.16, 0.05	−0.16, 0.06	0.00	0.00	NS as predicted (T3)
EP_sec—H3: SR → EP											
EP_sec	CSR_mean	H3: SR → EP	0.35	<0.001	0.06	0.26	0.25, 0.44	−0.05, 0.17	0.18	0.01	—
	ASR_mean		0.26	<0.001	0.07	0.23	0.17, 0.36	−0.04, 0.17	—	—	
PD_mean—H4: AP → PD											
PD_mean	AP_mean	H4: AP → PD	0.31	<0.001	0.10	0.06	0.20, 0.42	−0.00, 0.21	0.30	0.02	—
	SN_mean		0.30	<0.001	0.06	0.29	0.19, 0.41	−0.05, 0.17	—	—	
EP_sec—Full model → EP											
EP_sec	AP_mean	Full model → EP	0.16	0.00	−0.02	0.67	0.05, 0.27	−0.13, 0.08	0.20	0.01	—
	CSR_mean		0.27	<0.001	0.06	0.26	0.16, 0.38	−0.05, 0.17	—	—	
	ASR_mean		0.27	<0.001	0.06	0.24	0.18, 0.37	−0.04, 0.17	—	—	
PD_mean—Full model → PD											
PD_mean	SN_mean	Full model → PD	0.24	<0.001	0.06	0.31	0.13, 0.34	−0.05, 0.16	0.41	0.02	—
	AP_mean		0.25	<0.001	0.10	0.07	0.15, 0.35	−0.01, 0.21	—	—	
	SS_mean		−0.35	<0.001	−0.09	0.09	−0.44, −0.26	−0.20, 0.02	—	—	

Standardised OLS. T0 = baseline (natural cultural mechanism); T3 = post-intervention. Range restriction may attenuate T3 *β*. *R*^2^ on first predictor row per outcome block. Green = *p* < 0.05 in predicted direction at T3.

## 4 Discussion

### 4.1 Validation of the dual-pathway model at baseline: how cultural norms shape motivation and health

The baseline path analysis provided strong support for the proposed dual-pathway model linking shame norms, motivational regulation, and athletic outcomes under naturalistic cultural conditions. Consistent with the latent structural equation model reported in Table 6, the observed-score estimates in Table 9—reported here for their interpretability and associated confidence intervals—showed the same pattern. Supporting H1, shame norms emerged as the strongest predictor of achievement pressure (observed-score *β* = 0.57, 95% CI [0.49, 0.66], *p* < 0.001, *R*^2^ = 0.33; corresponding latent estimate *β* = 0.70, *p* < 0.001). Although these constructs were strongly correlated (*r* = 0.57), HTMT values among the focal constructs remained below the conventional 0.85 threshold (see Table 5), supporting their discriminant validity and indicating that shame norms capture culturally embedded group-level expectations surrounding failure, whereas achievement pressure reflects performance-oriented demands within the sport environment. This finding extends SDT-based motivational climate research by identifying shame norms as a culturally specific upstream antecedent of performance pressure rather than merely a parallel motivational construct. Supporting H2a, achievement pressure selectively predicted controlled self-regulation (observed-score *β* = 0.48, *p* < 0.001; latent *β* = 0.57, *p* < 0.001) but was not significantly associated with autonomous self-regulation (observed-score *β* = −0.06, *p* = 0.27; latent *β* = −0.08, *p* = 0.19). This non-significant coefficient does not confirm the null hypothesis; it only indicates that the expected AP-to-ASR association was not detected. To further evaluate whether this non-significant finding reflected insufficient evidence or support for the absence of a meaningful association, an additional Bayesian evidence analysis was conducted on the observed-score model. The Bayes factor supported the null model (BF10 = 0.060), indicating that the observed data were substantially more consistent with no meaningful AP-to-ASR association than with an alternative model assuming an effect. Therefore, the findings are consistent with the interpretation that culturally embedded pressure operates primarily through controlled rather than autonomous motivational processes. This pattern extends previous findings on performance climates by demonstrating a norm-based pathway through which collective shame expectations shape motivational regulation.

The performance pathway involving self-regulation and endurance outcomes requires cautious interpretation. Both controlled and autonomous self-regulation were significantly associated with baseline 5,000 m completion time (CSR: observed-score *β* = 0.35, latent *β* = 0.38; ASR: observed-score *β* = 0.26, latent *β* = 0.26; all *p* < 0.001), but because higher completion time indicates slower performance, these positive coefficients do not represent performance enhancement. Instead, the associations likely reflect the influence of accumulated training demands, competitive level, and physiological adaptation, suggesting that motivational regulation may function partly as an indicator of intensive athletic involvement rather than a direct determinant of performance. Supporting H4, shame norms and achievement pressure independently predicted psychological distress in the full observed-score model that included social support as a substantive covariate rather than a CMB marker (SN: *β* = 0.24; AP: *β* = 0.25; SS: *β* = −0.35; all *p* < 0.001, *R*^2^ = 0.41), and the corresponding latent estimates showed the same pattern (SN: *β* = 0.26; AP: *β* = 0.25; SS: *β* = −0.42; both *p* ≤ 0.002). Taken together, the findings demonstrate that the same cultural pressure system can simultaneously sustain effort through controlled regulation while generating psychological costs, highlighting the dual nature of shame-based motivational environments in Chinese competitive sport.

### 4.2 Post-intervention path attenuation: range restriction or ACT-mediated decoupling?

The post-intervention path analysis revealed a substantial attenuation of the dual-pathway structure from T0 to T3. Specifically, the SN → AP association declined from an observed-score *β* = 0.57 to *β* = 0.13 (*p* = 0.02; corresponding latent estimates: *β* = 0.70 at T0 to *β* = 0.15 at T3, *p* = 0.02), while the AP → CSR and CSR → EP pathways became non-significant in both the observed-score and latent models, and the AP → PD pathway was reduced to a marginal level (observed-score *β* = 0.10, *p* = 0.06; latent *β* = 0.11, *p* = 0.09). Although this pattern suggests that the baseline cultural pressure mechanism was less strongly expressed after the intervention, the attenuation should not be interpreted as definitive evidence that the underlying motivational processes disappeared. One plausible explanation is range restriction: by T3, shame norms and achievement pressure had both declined (SN: *d* = −0.31; AP: *d* = −0.27), reducing variance in the predictors and mechanically weakening standardized associations. Such attenuation is common in pre-post designs where interventions reduce variability in theoretically important constructs (Hamaker et al., 2015; Lucas, 2023).

A theoretically important finding is that controlled self-regulation increased rather than decreased following the ACT-informed programme (M = 3.48 to 3.85, *d* = 0.50, *η*^2^*p* = 0.13), despite the expectation that psychological flexibility training would reduce reliance on controlled motives. This unexpected pattern may reflect several non-exclusive mechanisms. First, increased metacognitive awareness may have enhanced athletes’ recognition and reporting of controlled motives rather than producing a genuine increase in maladaptive regulation. Second, contextual factors, including ongoing competitive demands and training pressures, may have maintained external performance obligations during the intervention period. Third, athletes may have reinterpreted introjected regulation as meaningful commitment within their sporting identity rather than solely as psychological burden, consistent with SDT’s view that introjection occupies an intermediate position between controlled and autonomous motivation (Deci and Ryan, 2000). Importantly, the increase in CSR occurred alongside reductions in psychological distress (*d* = −0.43), burnout (*d* = −0.39), perceived exertion (*d* = −0.32), and improved endurance performance (*d* = −0.46), suggesting that the intervention may have altered the psychological consequences of controlled motivation rather than simply reducing its presence. Consistent with this interpretation, High-SN athletes showed substantially greater reductions in achievement pressure and distress than Low-SN athletes (both BF_10_ > 1,000), and improvements in psychological outcomes and performance were maintained at follow-up. Therefore, the most cautious interpretation is that T3 attenuation reflects a combination of statistical range compression, altered interpretation of motivational processes, and possible motivational decoupling. Given the single-arm design, the relative contribution of each mechanism cannot be determined, and the baseline model remains the strongest evidence for the culturally embedded motivational pathway operating under naturalistic conditions.

### 4.3 The positive CSR–performance association: training history, task type, and the motivational bind

The positive association between controlled self regulation and endurance performance at baseline (observed-score *β* = 0.35, 95% CI [0.25, 0.44], *p* < 0.001; corresponding latent estimate *β* = 0.38, *p* < 0.001) represents the most theoretically provocative finding in the present dataset because it runs counter to the canonical SDT prediction that controlled regulation is associated with poorer performance than autonomous regulation on complex and sustained tasks. Three explanations, which are not mutually exclusive, deserve consideration, and each carries different implications for theory and practice. The first is a task type account. The 5,000 m run is a high volume and repetitive endurance task whose outcome is determined primarily by accumulated training load and aerobic adaptation rather than by creative problem solving, tactical flexibility, or in competition decision making, which are the domains in which the performance gap between autonomous and controlled regulation is most consistently observed in the SDT literature (Ntoumanis and Moller, 2025; Reinebo et al., 2024). On highly automatised and volume dependent tasks, the distinction between controlled and autonomous regulation may matter less for short term performance because the most immediate determinant of performance is physiological capacity built up over months or years, not the motivational state operating at a single assessment point. Put differently, CSR may help sustain the training volume needed to build aerobic fitness while simultaneously undermining the psychological need satisfaction that protects long term well being, which is exactly what the health cost pathway captures. The second, and arguably more important, explanation is confounding by training history. Athletes with longer competitive experience are likely to have accumulated greater training loads under the predominantly controlled motivational climate of the Chinese national sport system, which may produce both higher CSR scores, through the gradual internalisation of external performance obligations into introjected regulation, and faster 5,000 m times through superior physiological development.

In the present sample, training experience ranged from 1 to 11 years (M = 5.0, SD = 2.2; see Table 1), and this variable was not included as a covariate in the structural models reported here; consequently, the extent to which training history accounts for the observed CSR-to-EP association cannot be determined from the present analyses, and this remains an open empirical question rather than one that sensitivity analyses have already resolved. The third explanation is a temporal asymmetry between motivational quality and performance outcomes. Controlled regulation may sustain short term behavioural output, such as completing training sessions and maintaining effort under social evaluation pressure, while its costs accumulate more slowly through progressive need frustration, burnout, and psychological distress. As a result, a cross sectional or even short window path analysis may capture the performance benefit before the cost to well being becomes large enough to impair performance. This interpretation is directly supported by the dual pathway structure of the present findings. The same athletes who showed high CSR also showed elevated psychological distress (*r* = 0.19, *p* < 0.001; Table 4) and burnout (*r* = 0.22, *p* < 0.001), suggesting that the performance benefit and health cost of controlled regulation occur at the same time rather than in sequence, which is precisely the motivational bind that the dual pathway model was designed to capture. Taken together, the most defensible conclusion is that the positive CSR to EP association reflects a context specific phenomenon produced by the joint influence of task type, potential confounding by training history, and the temporal structure of motivational costs, rather than evidence that controlled regulation is genuinely performance enhancing. Future research should test this interpretation directly by including training years, and other indicators of accumulated training load, as covariates in structural models—an analysis the present study was not able to conduct—by extending the follow up window to detect delayed performance costs of controlled regulation, and by replicating the design in sport contexts where performance depends more directly on autonomous motivational processes, such as tactical decision making or skill execution under competitive pressure.

### 4.4 Cross-lagged associations: directional evidence and its boundary conditions

The cross lagged partial correlation results provided time ordered evidence for the proposed directional relationships, although the pattern of significance across waves also revealed important boundary conditions that require careful interpretation. At the T1 to T2 transition, the theoretically predicted forward paths were significant. Shame norms prospectively predicted achievement pressure (*r* = 0.16, 95% CI [0.05, 0.26], *p* = 0.004), achievement pressure predicted both controlled self regulation (*r* = 0.15, 95% CI [0.05, 0.26], *p* = 0.004) and psychological distress (*r* = 0.20, 95% CI [0.10, 0.30], *p* < 0.001), and controlled self regulation predicted endurance performance (*r* = 0.11, 95% CI [0.00, 0.21], *p* = 0.04), together constituting a temporally ordered chain consistent with the dual pathway model operating within a 2 week lag interval. Importantly, the reverse paths did not persist into the later intervention period. Controlled self regulation predicting achievement pressure attenuated from *r* = 0.15 at T1 to T2 to small, non-significant, and slightly negative values at later transitions (*r* = −0.04 and −0.03 at T2 to T3 and T3 to T4, respectively), and psychological distress predicting achievement pressure attenuated from *r* = 0.15 and 0.18 at T1 to T2 and T2 to T3 to r = 0.06 at T3 to T4, supporting the directional specificity of the proposed model in the sense that cultural pressure appears to precede and predict motivational and affective responses rather than the reverse (see Table 8). At the same time, most of the forward paths attenuated to non significance by T2 to T3 or T3 to T4, with one notable exception: the AP → PD path remained significant at T2 to T3 (*r* = 0.16, *p* = 0.00) and only attenuated to non-significance at T3 to T4 (*r* = 0.07, *p* = 0.22), indicating that the achievement-pressure-to-distress link was comparatively more persistent across the intervention window than the other three forward paths. This wave specific pattern also requires explanation.

Two mechanisms are likely to be operating. First, the intervention delivered its most intensive content between T1 and T3, and the resulting motivational changes may have disrupted the natural covariance structure between adjacent wave scores captured by the cross lagged partial correlations, not because the causal mechanism ceased to operate, but because within person fluctuations around each participant’s stable level became less predictable from the preceding wave once active psychological skills training was underway. Second, even at T1 to T2, the cross lagged effect sizes were small, ranging from *r* = 0.11 to 0.20, which is consistent with the expectation that prospective within person associations will be weaker than between person correlations once autoregressive stability is controlled, given that the between person component of the SN to AP relationship was substantially larger at baseline (*r* = 0.57 at T0) than any wave to wave fluctuation, and that this stable individual difference, rather than the within person dynamics, carries most of the structural signal in the baseline regression analysis. A full random intercept cross lagged panel model (Hamaker et al., 2015; Mulder and Hamaker, 2021), which would have formally separated between person and within person variance, was not estimable in the present design because the analysis relied on composite rather than latent indicators. The present cross lagged partial correlations therefore represent a conservative and methodologically transparent approach to directional inference, although one that remains limited by the inability to cleanly distinguish stable between person differences from dynamic within person processes. Taken together, the cross lagged findings contribute a specific but bounded inferential value to the overall evidence base. They show that prior wave levels of shame norms prospectively predicted subsequent achievement pressure after controlling for autoregressive stability at the T1 to T2 interval, thereby providing time ordered support for the directional hypothesis beyond what cross sectional correlation alone can establish, while also making clear that the dominant signal in the data lies in stable between person variance rather than dynamic within person fluctuation, a distinction that future measurement burst designs with shorter intervals between waves could decompose more precisely.

### 4.5 Subgroup differences: cultural vulnerability, intervention responsiveness, and the performance–wellbeing disconnect

The median split subgroup analysis revealed a coherent pattern of baseline disadvantage and differential intervention responsiveness that substantially enriches the interpretation of the main effect findings. At T0, High SN athletes presented with a profile consistent with acute cultural motivational vulnerability. Relative to Low SN athletes, they reported significantly greater achievement pressure (*d* = 1.06, BF10 > 1,000), controlled self regulation (*d* = 0.88, BF10 > 1,000), psychological distress (*d* = 0.92, BF10 > 1,000), perceived exertion (*d* = 0.68, BF10 > 1,000), and burnout (*d* = 0.64, BF10 > 1,000), with Bayes factors uniformly exceeding 1,000 and thus indicating decisive rather than merely significant evidence for all five differences. At the same time, they ran more slowly at baseline (*d* = 0.43, BF10 = 224.7), suggesting that the cultural motivational bind identified in the full sample path analysis was concentrated most strongly in the subgroup exposed to the greatest normative shame pressure (see Table 7). Notably, autonomous self regulation did not differ between groups at T0 (*d* = −0.18, *p* = 0.08, BF10 = 0.5), and the Bayes factor provided positive evidence for the null. This reinforces the double dissociation observed in the path analysis and suggests that shame norms selectively elevate the controlled motivational profile without suppressing autonomous motivation, a pattern consistent with the SDT prediction that externally contingent pressure activates introjected and external regulation while leaving identified and intrinsic motivation structurally intact (Deci and Ryan, 2000; Shannon et al., 2023).

Following the intervention, High SN athletes demonstrated substantially larger improvements on the psychosocial outcomes most directly targeted by the ACT-informed multimodal programme. Reductions in achievement pressure (Δ = −0.46 vs. +0.04, *d* = −0.66, BF10 > 1,000), psychological distress (Δ = −0.62 vs. −0.12, *d* = −0.60, BF10 > 1,000), and perceived exertion (Δ = −0.74 vs. −0.08, *d* = −0.53, BF10 > 1,000) were all markedly greater in the High SN group, while burnout reduction was also significantly larger (Δ = −0.48 vs. −0.14, *d* = −0.43, BF10 = 144.3). This convergent pattern strongly suggests that the intervention was most therapeutically active for athletes whose baseline cultural pressure profile gave the ACT mechanisms the most to work on. By T3, the Bayesian evidence for group differences in autonomous self regulation, perceived exertion, burnout, and social support had reversed to favour the null (all BF10 < 1), indicating that the High SN group’s initial psychosocial disadvantage had been largely equalised. This finding is consistent with the dose response logic of ACT, whereby the programme’s defusion and values clarification components produce proportionally greater psychological flexibility gains in individuals who begin with higher levels of experiential avoidance and shame based cognitive fusion (Ronkainen et al., 2025; Hayes et al., 2012). The most theoretically significant subgroup finding, however, concerns endurance performance. Despite the High SN group showing substantially larger improvements in psychological distress, perceived exertion, and burnout, the two groups showed only a small difference in the magnitude of their performance gains (Δ = −57.8 s vs. −35.3 s, *d* = −0.22, *p* = 0.04). Although this difference reached conventional statistical significance, the associated Bayes factor (BF10 = 0.8, i.e., BF01 = 1.25) fell within the anecdotal range and did not provide strong evidence for either performance equivalence or a genuine group difference; the evidence for a meaningful disparity in performance gains between groups therefore remains inconclusive rather than settled in either direction. This performance-wellbeing disconnect, whereby psychological health improvements of considerable magnitude did not translate into a clearly larger objective performance gain within the six to eight week intervention window, is important in two respects. First, it cautions against assuming that short term psychological skills training will generate commensurate performance returns within a single competitive season, which is consistent with meta analytic evidence showing that psychological interventions tend to produce only modest average effects on objective performance even when mental health outcomes improve substantially (Reinebo et al., 2024). Second, it suggests that the performance benefits of reducing shame based pressure may unfold over a longer time horizon than the psychological benefits, potentially because reduced burnout related disruption to training accumulates across multiple seasons rather than across a matter of weeks, a possibility that will require prospective follow up designs extending well beyond the 4 week T4 assessment used here.

### 4.6 Limitations and future directions

The present study advances understanding of cultural motivational mechanisms in Chinese competitive sport, but several limitations constrain the strength of its conclusions and point toward specific directions for future investigation. The most consequential limitation is the absence of a no intervention control group, which precludes causal attribution of the observed pre to post changes to the ACT-informed multimodal programme and prevents formal separation of intervention effects from range restriction, maturation, and seasonal training variation. This limitation arose from ethical constraints, specifically the reluctance to withhold a structured psychological skills programme from competitive athletes during an active training season, and from the practical unavailability of a matched comparison cohort across the three recruitment sites, but it nonetheless represents the primary boundary on the inferential scope of the T3 findings. A recently published ACT based randomized controlled trial in athletes provides a useful methodological template for future research (Ronkainen et al., 2025), suggesting that a fully randomized design matching the present programme structure, including the ACT based group format, the 6 to 8 week delivery window, and the dual assessment of psychosocial and objective performance outcomes, would enable the causal questions that the present single arm design cannot address. A second limitation concerns potential confounding by training history in the CSR-to-EP association discussed in Section 4.3. Training experience, weekly running distance, and competitive level were measured (see Table 1), but were not included as covariates in the primary structural models reported here; consequently, whether training history, training quality, physiological capacity, coaching environment, or accumulated competitive exposure account for part or all of the observed CSR-to-EP association cannot be determined from the present analyses. This confound should be addressed in future work by including training years, weekly training volume, and competitive level as covariates within the SEM framework, and by extending the follow up window to 12 to 24 months to test whether the performance advantage associated with CSR erodes as the health cost pathway accumulates through progressive burnout and need frustration. Third, the exclusive reliance on self report measures for all psychological constructs, despite the procedural and statistical controls implemented for common method bias, leaves open the possibility that method specific variance inflated the observed associations. This concern is particularly relevant for the SN to AP and AP to PD paths, where both predictor and outcome share the same response format and measurement context. Future studies would benefit from incorporating physiological indices of psychological stress, including cortisol reactivity, heart rate variability, and sleep quality, as objective complements to self report measures, and from assessing shame norm perception through peer report or coach rated team climate instruments to establish convergent validity for the SSNS beyond its self report form.

Fourth, the sample was drawn exclusively from Chinese university athletics programmes across three cities, which limits generalisability in two respects. Within culture, the findings may not extend to elite national level athletes or to recreational runners for whom institutional shame norm structures are less pervasive. Across cultures, the dual pathway model has not yet been tested in other Confucian heritage contexts such as South Korea and Japan, nor in individualist cultural settings where normative shame is expected to be less motivationally salient. Comparative research across these contexts would directly test the cultural specificity implied by the present findings. Fifth, the Sport Shame Norms Scale was developed specifically for this study and, although it demonstrated acceptable internal consistency and theoretically coherent correlational patterns, it has not yet been validated against external criteria, examined for temporal stability across competitive seasons, or tested for measurement invariance across cultural groups. Further validation work is therefore necessary before the scale can be confidently applied in cross cultural or applied screening contexts. Finally, the 4 week T4 follow up window is insufficient to determine whether the psychological gains observed at T3 translate into durable performance improvements across a full competitive season, whether the equalisation of High SN and Low SN groups on psychosocial outcomes is maintained once normative pressure re intensifies, and whether the motivational decoupling suggested by the T3 attenuation persists over time. These questions require prospective designs spanning at least one full competitive season, ideally longer, with the inclusion of booster sessions to test whether ACT effects require periodic reinforcement to be sustained within a high pressure cultural environment. Sixth, because baseline (T0) records for the 43 participants who withdrew before completing all five waves were not retained in the verified analytic data files, a formal comparison between completers and non-completers on baseline characteristics could not be conducted (see Section 2.1). Given that psychologically burdened, younger, or less experienced athletes may be differentially likely to withdraw from a multi-wave intervention study, the possibility of non-random attrition cannot be ruled out. This remains an unverified but plausible source of bias affecting the generalisability of the complete-case results, and future studies should retain complete baseline records for all recruited participants, including those who subsequently withdraw, to enable a formal evaluation of selection bias. Seventh, the sample included a small proportion (6.1%, *n* = 21) of national-club athletes alongside the predominantly university-varsity sample (49.6%) and other competitive levels (see Table 1). These national-club athletes may differ from the rest of the sample in physiological capacity, training exposure, and motivational profile, introducing a potential source of internal heterogeneity distinct from the cross-sample generalizability concern discussed above. Although competitive level was measured, it was not included as a covariate in the primary structural models reported in Sections 3.2 and 3.6 (see also Section 4.3), and this potential heterogeneity has therefore not been formally tested. Future research should consider stratifying analyses by competitive level, or conducting a robustness check that excludes national-club athletes, to determine whether the reported associations—particularly the CSR-to-EP path—hold consistently across athletes of differing competitive standing.

## Data Availability

The raw data supporting the conclusions of this article will be made available by the authors, without undue reservation.
